# High-quality draft genome sequence data of *Levilactobacillus brevis* 3LB isolated from fermented milk koumiss

**DOI:** 10.1016/j.dib.2026.112985

**Published:** 2026-06-18

**Authors:** Diana Kurmangali, Gulyaim Abitayeva, Zhandarbek Bekshin

**Affiliations:** Republican Collection of Microorganisms LLP, Astana 010000, Kazakhstan

**Keywords:** *Levilactobacillus brevis*, Lactic acid bacteria, Genome sequence, Genome annotation, Functional annotation, Safety assessment, Probiotic potential

## Abstract

This study presents the high-quality draft genome sequence of *Levilactobacillus brevis* 3LB (*L. brevis* 3LB), a strain derived from a fermented milk koumiss from the Akmola region of the Republic of Kazakhstan. This strain is stored in the National Depository of Kazakhstan, Republican Collection of Microorganisms LLP, under the identifier *L. brevis* 3LB B-RKM 0546. Genomic DNA sequencing was performed on the Illumina MiSeq platform, and *de novo* genome assembly was carried out using SPAdes Genome Assembler v3.15.5. The assembled draft genome was 2307,466 bp in size, with 150x coverage, a GC content of 46.01%, an L50 value of 7, and an N50 value of 168,338 bp. The genome consists of 63 contigs and contains 2260 coding genes, as well as 61 transfer RNA (tRNA) genes, 7 ribosomal RNA (rRNA) genes, and 2 CRISPR loci. Functional analysis of the 3LB strain genome revealed the dominance of genes related to metabolism, information storage and processing, and cellular processes, providing a comprehensive understanding of the biological potential of bacteria by elucidating their biochemical reactions, metabolic pathways, and mechanisms of adaptation to the environment. According to GO analysis, these categories contain the highest number of genes (786, 549, and 361, respectively). Among the COG functional categories, transcription (228), translation and ribosome biogenesis (166), and carbohydrate metabolism (166) stand out. Cellular motility was the least represented (8 genes). KEGG analysis also confirmed the dominance of metabolism (446) and genetic information processing (296). RAST annotation further emphasized the importance of carbohydrate metabolism (19.82%), protein synthesis (16.37%), and amino acid synthesis (9.85%). CAZymes analysis identified 52 genes encoding enzymes involved in carbohydrate modification, with glycoside hydrolases (26) and glycosyltransferases (20). A genomic safety analysis of the *L. brevis* 3LB strain revealed that it is not a human pathogen, as it does not contain virulence genes, and the probability of structural pathogenicity is extremely low (0.0314). Although three incomplete phage regions and genes with low identity to vanT and nimA (requiring further study) were detected *in silico*, no known antibiotic resistance genes were detected. The absence of plasmids also confirms its safety. Notably, the strain possesses two high-fidelity CRISPR arrays, providing protective mechanisms. Furthermore, three secondary metabolite regions capable of synthesizing compounds with potential antibacterial activity (a class IV lanthanide peptide, a terpene precursor, and a polyketide) were identified, giving the strain a competitive advantage. These genome mining results indicate the biotechnological significance, safety, and probiotic potential of the novel strain *L. brevis* 3LB. The data presented here provide a foundation for further research into the probiotic activity of strain 3LB, both *in vitro* and *in vivo*, as well as its potential applications in food industry and biomedicine.

Specifications Table

**Table:** 

Subject	Biology
Specific subject area	Biological sciences, Bioinformatics, Microbiology, Agricultural Sciences
Type of data	Table and Figure
Data collection	DNA was extracted from a fresh overnight culture of *L. brevis* 3LB via standard phenol-chloroform purification. Whole-genome sequencing was then conducted on an Illumina MiSeq platform (Illumina, USA). The resulting genome was assembled *de novo* into contigs using SPAdes v3.15.5, with assembly quality evaluated by QUAST v5.2.0 and contamination/errors checked by CheckM v1.2.4 (98.03% complete; 0.74% contaminated). Prokka was employed to generate annotated genomes from these contigs. Functional annotation was achieved through eggNOG-mapper version 2.1.12 (utilizing COG and KEGG databases) and independently via RAST. CRISPR loci were identified using CRISPRCasFinder, and CAZymes were identified using dbCAN3 (HMMER). ANI was calculated using the OrthoANI tool (EzBioCloud), and the phylogenetic tree was constructed using the maximum likelihood method using kSNP. Antibiotic resistance genes were identified using CARD 4.0.1, ResFinder 4.7.2, and NCBI AMRFinderPlus. Virulence factors were identified with VirulenceFinder v2.0, pathogenicity with PathogenFinder2 v0.5.0, and prophages were detected using Phatest. Plasmid replicons were analysed with PlasmidFinder v2.1, and secondary metabolite clusters were identified using AntiSMASH v8.0.4.
Data source location	Institute: Republican Collection of Microorganisms • City/Town/Region: Astana • Country: Kazakhstan
Data accessibility	Repository name: National Center for Biotechnology Information (NCBI) Data identification number for NCBI (accession number): JBSJDQ000000000, BioSample SAMN52842156, BioProject PRJNA1346845 Direct URL to NCBI data: https://www.ncbi.nlm.nih.gov/nuccore/JBSJDQ000000000, https://www.ncbi.nlm.nih.gov/biosample/52842156, http://www.ncbi.nlm.nih.gov/bioproject/1346845
Related research article	None

## 1 Value of the Data

• Genomic data provide information on the functional properties of the *L. brevis* 3LB strain isolated from traditional fermented milk (koumiss), making them valuable for research in food biotechnology and microbiology.
• Genomic data of *L. brevis* identify a cluster of secondary metabolite synthesis (lantibiotics, polyketides, terpenes), which may provide additional antimicrobial activity and competitive advantages in fermentation processes.
• The genomic data of *L. brevis* 3LB expand our understanding of the species' genetic diversity, including its adaptation to the specific conditions of lactic acid fermentation.
• Genomic data demonstrate the absence of genes associated with virulence and antibiotic resistance, confirming the safety and genetic stability of the strain and making it a promising candidate for use in food and pharmaceutical products.

## 2 Background

*Levilactobacillus brevis (L. brevis)* is a gram-positive, heterofermentative lactic acid bacterium (LAB) with GRAS status [1]. The probiotic potential of *L. brevis* strains is being actively investigated, ranging from their survival in the harsh environment of the gastrointestinal tract to their therapeutic effects in cancer [[2], [3], [4]]. The application of new probiotic strains, however, requires thorough confirmation of their safety [5]. Regulatory authorities, such as the European Food Safety Authority (EFSA), emphasize the critical need to identify antibiotic resistance (AMR) profiles [6]. The research indicates that some probiotics may act as hidden reservoirs of AMR genes capable of horizontal transfer via mobile genetic elements (plasmids, transposons) [7]. In this regard, genomic data analysis contributes to understanding the genetic diversity, genotype, and functional characteristics of strains, as well as to excluding the presence of mobile resistance genes and confirming the genetic stability of strains prior to their introduction into production.

The study of the new *L. brevis* 3LB strain, isolated from a unique ecological niche (traditional koumiss), reveals specific probiotic properties determined by the conditions of natural fermentation of mare’s milk with high acidity [8]. *In vitro* testing of strain 3LB demonstrated antimicrobial activity against pathogens such as *Candida albicans, E. coli, Salmonella typhimurium* and *Staphylococcus aureus* (Table S1).

This study provides a high-quality draft genome sequence data of strain 3LB, including genomic characterization and functional annotations of genes associated with metabolic activity and adaptation. The demonstrated safety profile, together with the presence of genetic determinants for antimicrobial peptide synthesis, highlights *L. brevis* 3LB as a promising candidate for the development of new functional foods and potential biomedical applications.

## 3 Data Description

**Fig. 1:**
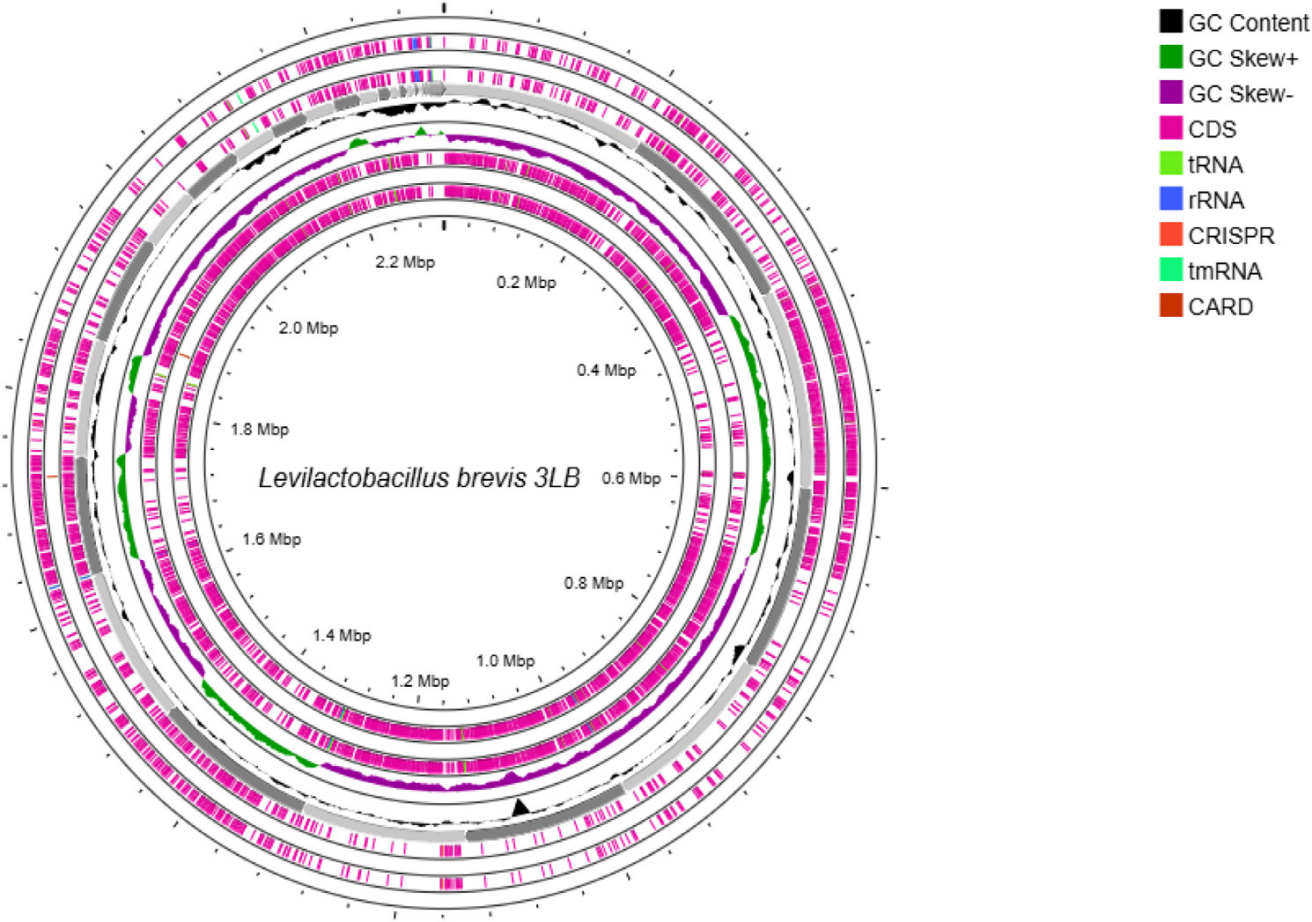
The genome map of the *L. brevis* 3LB bacterium was visualized using CGView (https://proksee.ca/, September 15, 2025). The genome information is presented as concentric circles, starting from the outer one: circle 1 shows annotated genes (CDS); circle 2 shows the percentage of guanine and cytosine (GC) content in the genome; circle 3 illustrates the deviation of GC/(G+C) content in the genome.

This article describes the whole-genome sequencing data of *L. brevis* 3LB, submitted to the National Center for Biotechnology Information (NCBI) database under the BioProject PRJNA1346845, BioSample SAMN52842156 and accession number DDBJ/ENA/GenBank JBSJDQ000000000. The size of the high-quality draft genome of the studied *L. brevis* 3LB strain was 2.3Mbp at 150x coverage (Fig. 1), which corresponds to typical indicators for representatives of the genus *L. brevis* [9].

The genome assembly consists of 63 contigs, the longest contig in the assembly is 208,011 bp. A total of 2260 coding sequences (CDS) were identified in the genome. 3LB contains a total of 69 RNAs: 61 transfer RNAs (tRNAs), 7 ribosomal RNAs (rRNAs), and 1 tmRNA molecule (Table 1).

**Table 1:** General genomic characteristics of *L. brevis* 3LB.

Feature	Value
Size of assembled genome, bp	2 307 466
CDS	2260
Number of contigs (total)	63
Number of contigs (>10,000 bp)	20
Number of contigs (>25,000 bp)	18
Number of contigs (>50,000 bp)	14
Length of the longest contig, bp	208 011
N50, bp	168 338
N90, bp	60 924
L50	7
L90	14
GC, %	46.01
CRISPR	2
Genome coverage	150x
tRNA	61
rRNA	7
tmRNA	1
Number of RNAs	69 (61 tRNA + 7 rRNA + 1 tmRNA)

**Fig. 2:**
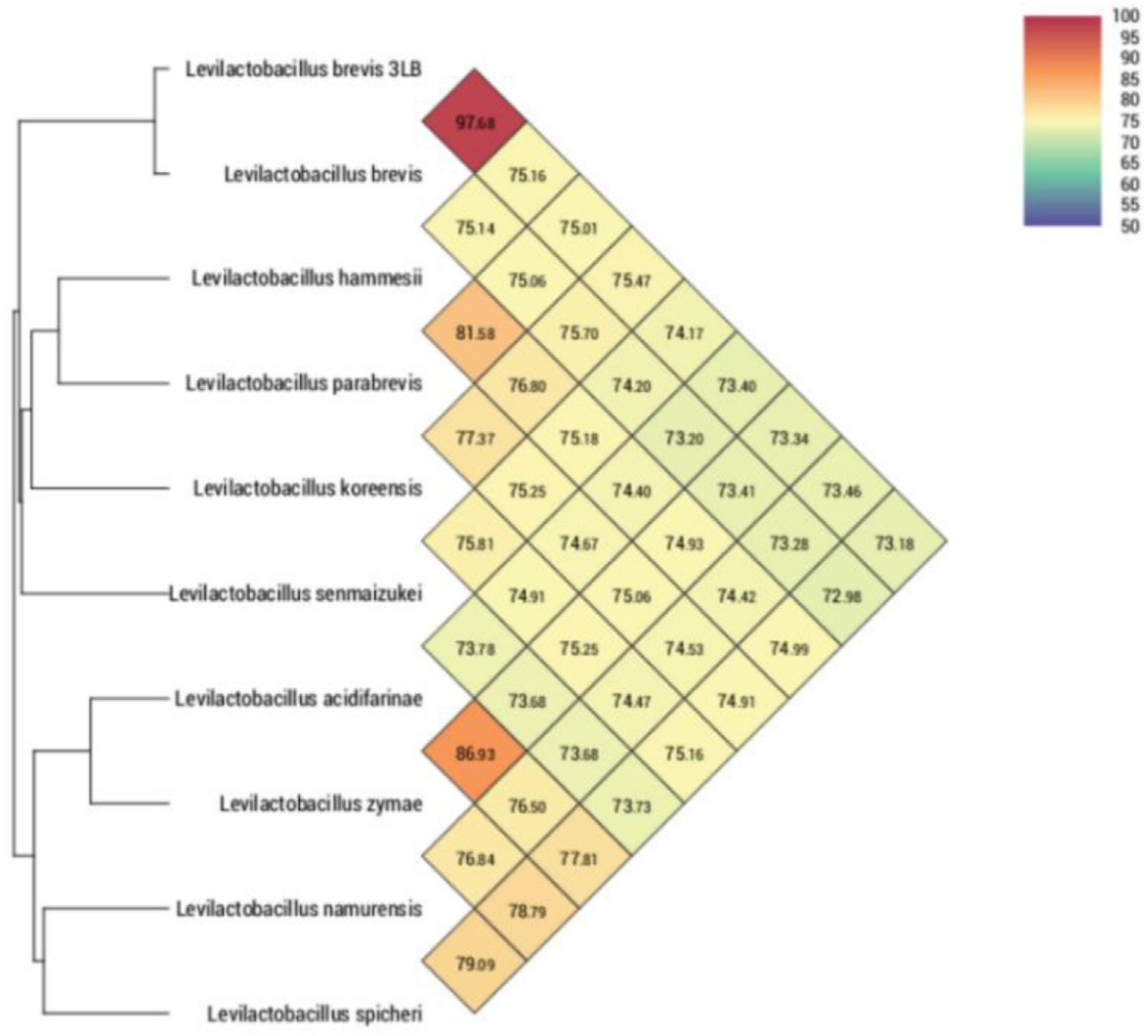
Using OAT software, OrthoANI values were calculated for the species *L. brevis* and its closely related nomenclatural species. The results are visualized on a heat map.

To assess the degree of genome similarity at the individual nucleotide level, the average nucleotide index (ANI) was calculated using the EZCloud online tool. Using the "average nucleotide identity" (OrthoANI) method, the genome of strain 3LB was analyzed and compared with representative species of the *Levilactobacillus* genera. The *L. brevis* UCCLB556 genome, obtained from NCBI under the identifier GCA_006228245.1, served as the reference genome for the calculations (Fig. 2). Both genomes were uploaded in FASTA QC format to the ANI calculator for subsequent analysis.

Fig. 2 shows that the “average nucleotide identity” results confirm the identity of strain 3LB as *L. brevis* UCCLB556 (GCA_006228245.1) with an accuracy of 97.68%, which is significantly higher than the threshold of 95–96% and indicates the presence of specific genomes [10].

**Fig. 3:**
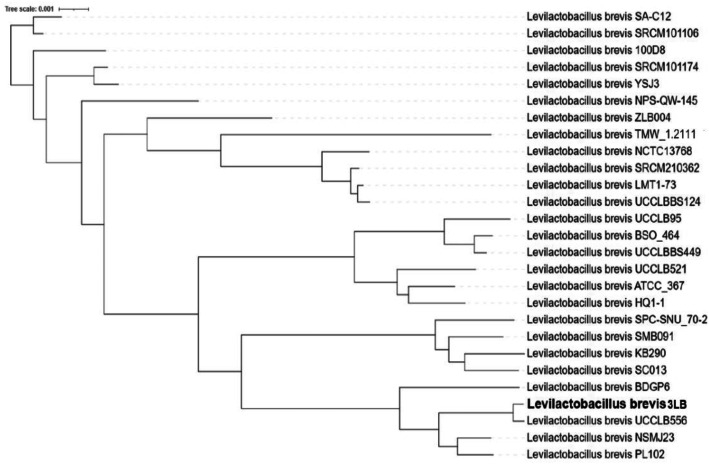
Phylogenetic tree showing the evolutionary relationships between strain *L. brevis* 3LB and 26 *L. brevis* strains.

The evolutionary relationship of strain 3LB is confirmed by its genomic data and comparison with the complete genomes of 26 *L. brevis* strains (Fig. 3; Table S2).

Based on phylogenetic analysis, the studied strain 3LB forms a branch with *the L. brevis* strain UCCLB556 (GCA_006228245.1, Netherlands, 2014) and is included in the same clade as the *L. brevis* strains NSMJ23 (GCA_014905055.1, South Korea, 2018) and *L. brevis* PL102 (GCA_024800685.1, South Korea, 2021).

### 3.1 Functional annotation of *L. brevis* 3LB

**Fig. 4:**
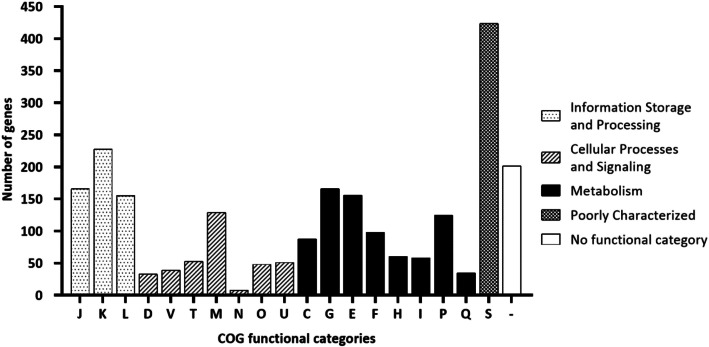
Distribution of annotated genes across functional COG categories. The classification is grouped into four main functional classes: Information Storage and Processing (J: Translation, ribosomal structure and biogenesis; K: Transcription; L: Replication, recombination and repair); Cellular Processes and Signaling (D: Cell cycle control, cell division, chromosome partitioning; V: Defense mechanisms; T: Signal transduction mechanisms; M: Cell wall/membrane/envelope biogenesis; N: Cell motility; O: Posttranslational modification, protein turnover, chaperones; U: Intracellular trafficking, secretion, and vesicular transport); Metabolism (C: Energy production and conversion; G: Carbohydrate transport and metabolism; E: Amino acid transport and metabolism; F: Nucleotide transport and metabolism; H: Coenzyme transport and metabolism; I: Lipid transport and metabolism; P: Inorganic ion transport and metabolism; Q: Secondary metabolites biosynthesis, transport and catabolism); Poorly Characterized (S: Function unknown). The symbol “–” indicates genes that could not be assigned to any known functional COG category.

**Fig. 5:**
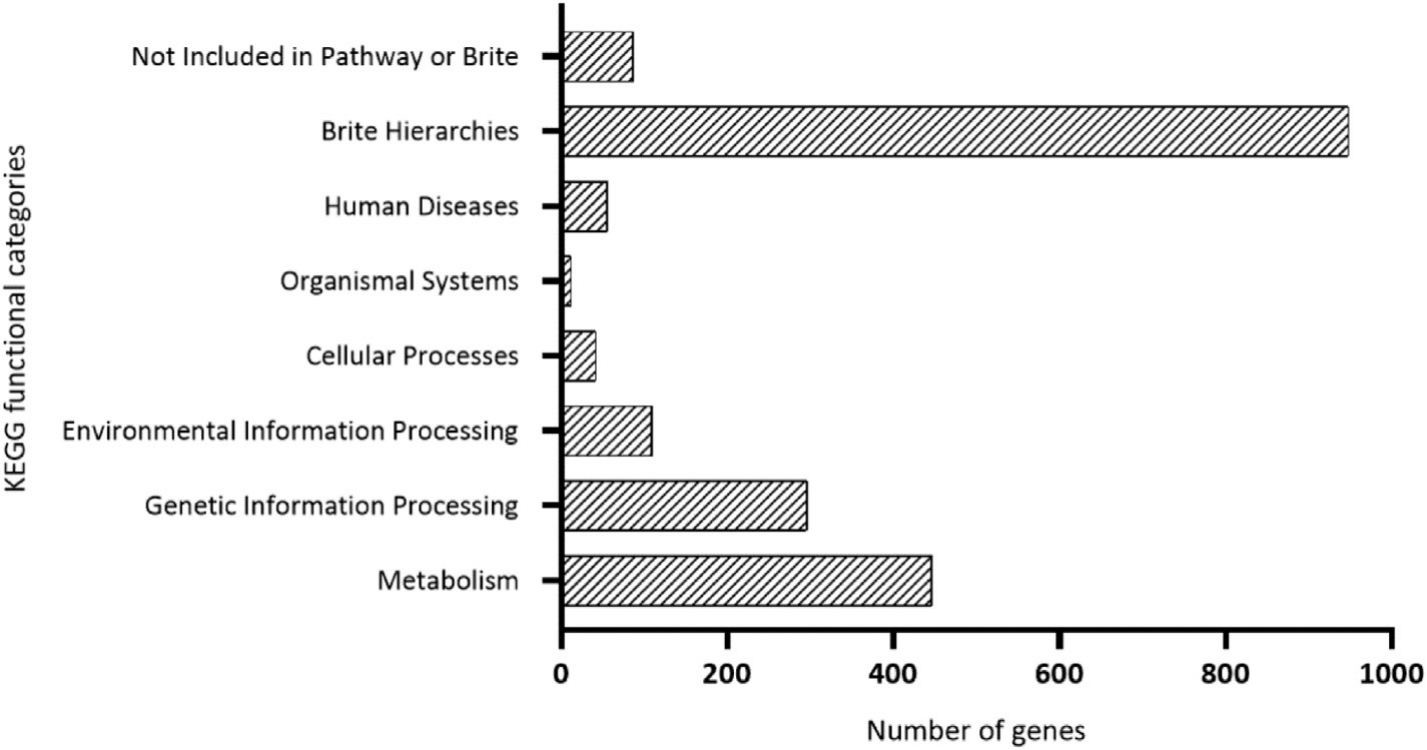
Distribution of annotated genome genes by functional categories of the KEGG database.

**Fig. 6:**
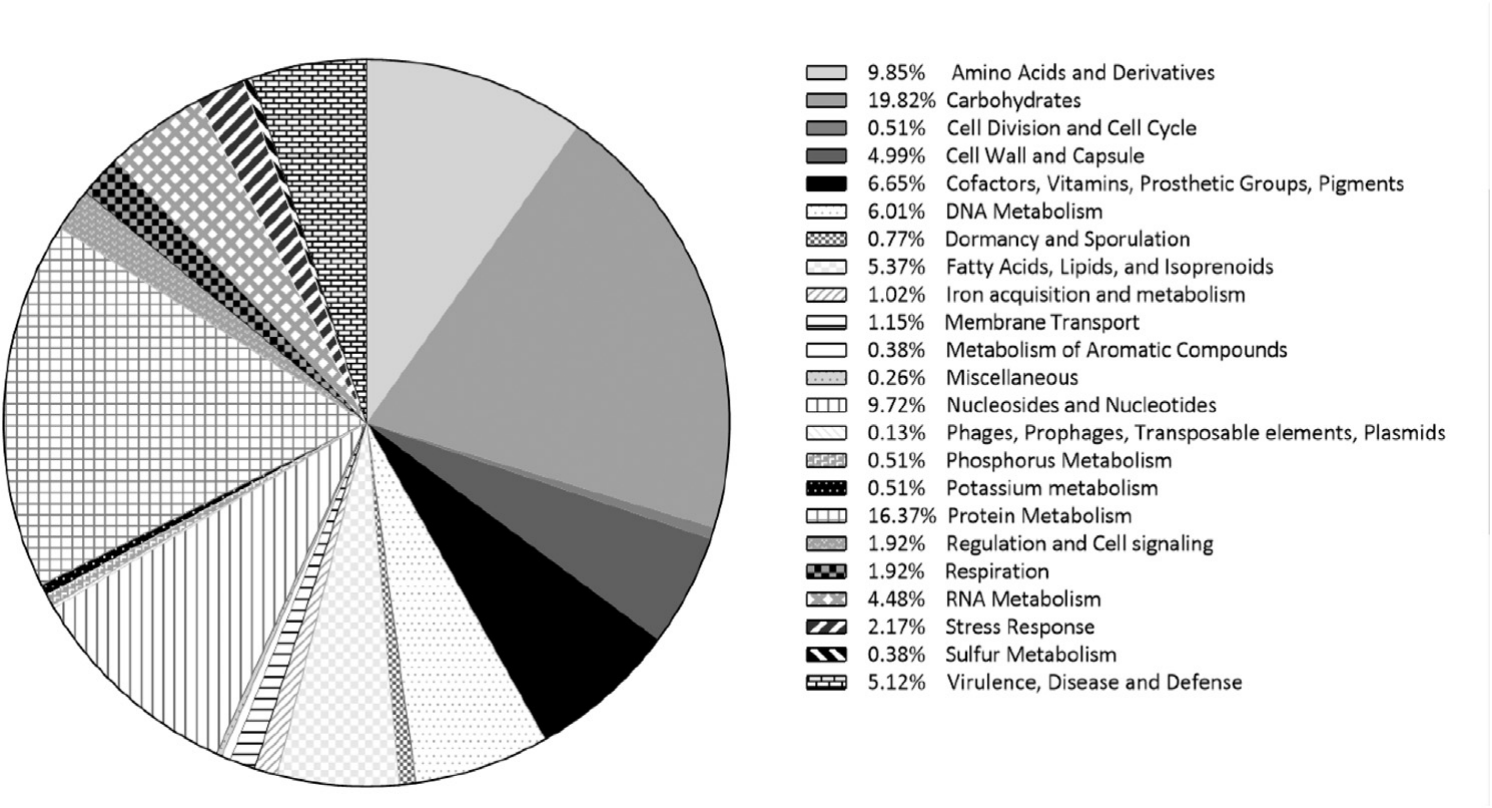
Functional classification of *L. brevis* 3LB genes based on RAST annotation results.

Functional annotation of COG (Fig. 4) and KEGG (Fig. 5) databases was performed using eggNOG (with recommended default settings), and analysis in the RAST system (Fig. 6) was carried out independently (Table S3-S5).

In 3LB strain, key gene categories from the GO data, such as metabolism (786 genes), information storage and processing (549 genes), cellular processes and signaling (361 genes), were found to account for a significant proportion of known genes.

The largest number of genes belongs to the S category (424 genes) of the COG database, indicating a corresponding proportion of genes with unknown constraints. High representation is also observed in the K category (228 genes) for transcription, J category (166 genes) for translation and ribosome biogenesis, and the G category (166 genes) for manifestations reflecting carbohydrate transport and metabolism in the genome. Fewer genes were identified in the N category (8 genes), which is associated with cell motility, which is characteristic of most *Lactobacillus* species.

KEGG annotation analysis of the 3LB strain revealed the following main categories: Brite Hierarchies (948 genes), indicating a hierarchical organization of genes. These are followed by categories reflecting key biological processes: Metabolism (446 genes) and Genetic Information Processing (296 genes). Furthermore, the following categories associated with other important functions were identified: Environmental Information Processing (109 genes), responsible for processing information about the environment; Human Diseases (55 genes), indicating genes associated with diseases; Cellular Processes (42 genes), reflecting cellular processes; Not Included in Pathway or Brite (87 genes), including elements not assigned to specific pathways or hierarchies; and Organismal Systems (12 genes), representing organismal systems. Thus, the annotations cover signaling pathways, diseases, cellular and organismal functions, as well as elements that do not have a clear classification.

An analysis of the predicted genes revealed that a significant proportion are related to key metabolic processes: the synthesis of carbohydrates (19.82%), proteins (16.37%), and amino acids and their derivatives (9.85%). A significant number of genes are also associated with functions including cofactors, vitamins, prosthetic groups, and pigments (6.65%). A more detailed distribution of the remaining genes by subsystem category is presented in Fig. 6.

Analysis of the 3LB genome using the dbCAN3 metaserver and the HMMER tool allowed us to identify and annotate enzymes involved in carbohydrate metabolism (CAZymes) (Table 2).

**Table 2:** The results of carbohydrate-active enzyme (CAZymes) annotation in the 3LB genome.

**CAZyme class**	CAZyme family (number of genes)
**Аuxiliary activities (AA)**	AA1 (1)
**Carbohydrate-binding modules** **(CBM)**	CBM91 (2)
**Carbohydrate esterases (CE)**	CE4 (2), CE9 (1)
**Glycoside hydrolases (GH)**	GH1 (1), GH13_31 (2), GH188 (1), GH2_10(1), GH2_3(1), GH25(4), GH3(1), GH30_9 (1), GH31_2 (1), GH31_3 (1), GH36 (1), GH43_11(2), GH43_26 (1), GH51_1 (2), GH65(3), GH73 (2), GH8 (1)
**Glycosyltransferases (GT)**	GT119 (3), GT2 (5), GT26 (1), GT28 (1), GT32 (1), GT4 (6), GT51 (2), GT8 (1)

Screening of the 3LB genome to identify carbohydrate-active enzymes revealed the presence of 52 genes belonging to five functional classes of CAZymes: glycoside hydrolases (GH, 26 genes), glycosyltransferases (GT, 20 genes), carbohydrate esterases (CE, 3 genes), carbohydrate binding modules (CBM, 2 genes), and auxiliary activities (AA, 1 gene). Among them, the most numerous families were GH25 (4 genes), GT4 (6 genes), and CE2 (2 genes).

In the context of bacterial adaptation, key enzymatic players are glycosyltransferases (GTs) and glycoside hydrolases (GHs). GTs are critical for the biosynthesis of surface polysaccharides, which form the external spatial defense of the bacterial cell (GT4, GT2). At the same time, GHs, represented by the GH25, GH65, and GH73 families, perform functions in remodeling the peptide glycan cell wall and promote the release of immunostimulatory molecular fragments [11].

### 3.2 Safety assessment of *L. brevis* 3LB

The genomic safety of the strain was examined through the application of multiple programs designed to detect virulence and antimicrobial resistance traits with implications for human or animal health. Subsequent analysis of the strain's genome utilizing ResFinder 4.7.2 (Table S6) and NCBI AMRFinderPlus did not reveal the presence of known antibiotic resistance genes. However, analysis using RGI 6.0.5 (CARD 4.0.1) identified genes similar to vanT and nimA (Table S7). Nevertheless, due to their low identity (31.82% and 48.15%, respectively), it can be concluded that these genes are unlikely to represent clinically relevant resistance. According to the 2024 guidelines of the EFSA, confirming the presence of antibiotic resistance genes requires a sequence identity of at least 70% and a sequence coverage of at least 90% [6]. At the same time, given the incompleteness of the genome assembly, the possibility of the existence of additional resistance genes cannot be completely ruled out, so the conclusions drawn from this interpretation require careful consideration and further research.

Analysis using VirulenceFinder v2.0 did not identify genes associated with virulence (Table S8). Analysis using the PathogenFinder2 v0.5.0 predictive tool showed that the *L. brevis* strain is not a human pathogen. The pathogenicity probability of the structure is 0.0314 (0.031), as no matches with pathogenic modules were found (0), while 273 matches with nonpathogenic modules were found (Table S9).

Analysis of the *L. brevis* 3LB genome revealed three incomplete regions associated with phages, but with low confidence (score <70). No regions corresponding to intact prophages were detected (score >90) (Table 3).

**Table 3:** Identification of prophages using Phatest in the *L. brevis* 3LB genome.

Region	Length	Completeness (score)	Region position	Most Common Phage
1	12Kb	incomplete (10)	NODE_3	PHAGE_Lactoc_lato_NC_004746(1)
2	14.7Kb	incomplete (60)	NODE_15	PHAGE_Entero_vB_EfaS_AL2_NC_042127 (3)
3	9Kb	incomplete(40)	NODE_20	PHAGE_Lactob_Lb_NC_047983(4)

Three putative phage regions, 9–14.7 kb in length, were identified in the genome, all of which were classified as incomplete. The longest region is located at NODE_15 (14.7 kb, score 60) and is associated with the phage PHAGE_Entero_vB_EfaS_AL2. The other regions are found at NODE_3 (12 kb) and NODE_20 (9 kb) and are related to the phages PHAGE_Lactoc_lato_NC_004746 and PHAGE_Lactob_Lb_NC_047983, respectively (Table S10).

None of the known families of replicon-associated plasmid sequences (Rep families: Rep1, Rep2, Rep3, RepL, RepA_N, Inc18, etc.) were detected in the genome using the PlasmidFinder v2.1 server (Table S11).

The 3LB genome was analyzed using the CRISPRCasFinder online tool, revealing the presence of clustered, regularly interspersed short palindromic repeats (CRISPR) (Table 4).

**Table 4:** Characterization of CRISPR cassettes in *L. brevis 3*LB.

Contig	Spacers	DRLength	CRISPR start	CRISPR end	Length	Array
NODE_7	6	28	22,168	22,561	394	R_AGACCACCCCTACATATGTGGGGAATAC S_TTGGTTCAAATGCTACAAGCTCGTTTTCAGCCA R_GGATCACCCCCACACGTGTGGGGAATAC S_ATACCTAGCTTTTTTCTATTCGACCAACTAATA R_GGATCACCCCCACACGTGTGGGGAATAC S_CACTAAGGCAGCTAAGCCGAAAAAGGCCATCTA R_GGATCACCCCCACATGTGTGGGGAATAC S_GAAAAATCTGTTGGGCTATAAGGCTACAGCAGA R_GGATCACCCCCACATGTGTGGGGAATAC S_ACAGTTGTTTGTGGAATATCTTGGTACTGTCCA R_GGATCACCCCCACATGTGTGGGGAATAC S_TTCATAAAAAAGCCGTCATTCCGCATTCAATGG R_GGATCACCCCCACACGTGTGGGGAATAC S_

NODE_11	5	28	27,540	27,872	333	R_GAAATGCCCCCACACCTGTGGGGTATAC S_GCTTAAAATCAAGCAGTCGTTGGGCTACGTCAA R_GGATCACCCCCACACCTGTGGGGAATAC S_TGACAACCAAAAAATATACGACTTTAACTTCTG R_GGATCACCCCCACACCTGTGGGGAATAC S_CGGAAAGTTTAAATCTATTAAAGACGCACAATA R_GGATCACCCCCACACCTGTGGGGAATAC S_CCGGTATGCGTATCGAAGAAGTCCCAACCAATG R_GGATCACCCCCACACCTGTGGGGAATAC S_GATGAATAAGAAAAGAAAACAAAATAATACGCA R_GGATCACCCCCACACCTGTGGGGAATAC S_

During the analysis of CRISPR systems, two high-confidence arrays (level 4) were identified. A 394-bp array (positions 22,168–22,561) was detected on NODE_7, characterized by the presence of six spacers and 28-bp repeats. The second array, located on NODE_11 (positions 27,540–27,872), is 333 bp long and contains five spacers with a 28-bp repeat.

**Fig. 7:**
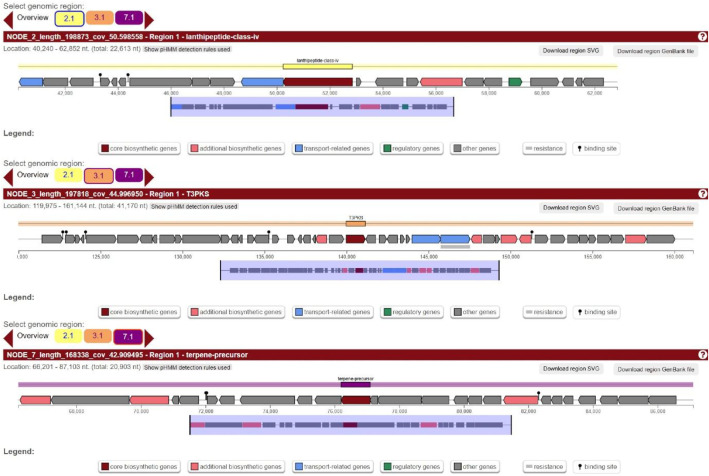
Annotation of secondary metabolite gene clusters of strain 3LB using antiSMASH 8.0.4.

Three secondary metabolite regions were identified using the relaxed stringency version of antiSMASH 8.0.4, potentially capable of synthesizing: lanthipeptide class IV, terpene precursor, and polyketide (T3PKS) (Fig. 7).

Of particular interest in the 3LB genome are the presence of a class IV lanthipeptide, a type III polyketide synthase (T3PKS), and a terpene precursor. The lanthipeptide cluster contains the ctg2_57 gene, located in the range 50,240–52,852 bp. It contains domains characteristic of serine/threonine protein kinases and lanthionine synthases (Pkinase, LANC_like). This indicates its involvement in post-translational modification of peptides, a key step in the synthesis of lantibiotics - compounds with antibacterial activity against Gram-positive bacteria [12]. Furthermore, the ctg3_140 gene, identified in the T3PKS cluster, contains a domain characteristic of enzymes synthesizing chalcone-like compounds linked to hydroxymethylglutaryl-CoA. Products of the T3PKS cluster may exhibit antimicrobial and fungicidal activity, inhibiting the growth of competing microorganisms [1,13]. Moreover, the ctg7_80 gene encodes polyprenyl synthase (PT_FPPS_ type), which is involved in the biosynthesis of terpene precursors, suggesting a role in the formation of isoprenoid compounds. Similarity to known terpene clusters and regulatory elements (CodY, LexA, PsrA) indicates potential involvement in the synthesis of biologically active terpenes and complex regulation of expression [14,15]. However, the absence of predicted key peptides and known analogs suggests that these clusters may be incomplete and require further research and in-depth study. The presence of secondary metabolites suggests that this strain is capable of providing competitive advantages and adapting to environmental conditions, protecting its ecological niche.

## 4 Experimental Design, Materials and Methods

### 4.1 Isolation of *L. brevis* 3LB strain and growth conditions

Koumiss is a fermented milk made from mare's milk, produced by fermentation. A koumiss sample was collected in the Akmola region (52° N 69° E, Kazakhstan) and delivered frozen to the laboratory. After incubation on De Man, Rogosa, and Sharp (MRS) selective medium (Condalab, Madrid, Spain) at 37 °C for 48 h, a pure isolate of *L. brevis* 3LB was obtained by streak plating [8]. The isolate obtained was stored at −80 °C in the LLP Republican Microorganism Collection under identification number B-RKM 0546.

### 4.2 Genomic DNA preparation and DNA sequencing

Genomic DNA from *L. brevis* 3LB strain was extracted from a new overnight culture cultivated in MRS broth; the cells were pelleted via centrifugation at 5000 g for 10 min at 4 °C. DNA extraction was performed using the GeneJET Genomic DNA Purification Kit (Thermo Fisher Scientific, USA), according to the manufacturer’s protocol. Additional DNA purification was performed by ethanol precipitation [16]. The quality and concentration of the isolated DNA were determined using a NanoDrop spectrophotometer (Thermo Fisher Scientific, USA). The concentration of the isolated DNA was 50 ng/μl, and the A260/A280 ratio was 1.825, indicating high purity of the samples and their suitability for further use. Gram staining, cell morphology, and 16S rRNA sequencing were the primary identification tools for these strains.

Whole genome sequencing was performed on the Illumina MiSeq platform (Illumina, USA) using the paired-end library preparation protocol (2 × 300 bp) and the Illumina DNA Prep Kit [17]. The sequencing output consisted of 300 bp reads, resulting in a 150x coverage depth and a total raw data yield of 2561,818 bp. Raw data were quality controlled using FastQC v0.12.1 [18]. Low-quality regions and adapters were then removed using SeqTK v1.4 (https://github.com/lh3/seqtk/releases, available April 15, 2024) and Sickle v1.33 (https://github.com/najoshi/sickle, available April 15, 2024). Clean sequences were assembled *de novo* in SPAdes v3.15.5 (https://github.com/ablab/spades/releases, available April 15, 2024) with a k-mer length of 127 and default settings. Assembly quality was assessed using QUAST v5.2.0 (https://github.com/ablab/quast, available April 15, 2024) and contamination and errors were checked using CheckM (https://github.com/Ecogenomics/CheckM, available April 15, 2024). The CheckM v1.2.4 program determined the genome completeness to be 98.03% and the contamination level to be 0.74% [19]. Based on the quality control results, it was decided to trim the reads by 20 bp from the 5′ end and 3 bp from the 3′ end, taking into account quality criteria of at least Q30 (accuracy >99.9%) and a minimum length of 100 bp. Calculation of average nucleotide identity (ANI) between industrial genomes was performed using the OrthoANIu tool on the EzBioCloud platform [20], based on the OrthoANI algorithm implemented with USEARCH [21]. The kSNP tool was used to create a phylogenetic tree based on whole-genome SNPs (wgSNPs), with an k-mer value of 19 obtained using the greatest likelihood method [22].

### 4.3 Genome annotation

Annotated genomes, represented by contigs, were obtained using Prokka [23]. The 26 complete genomes of the relevant species and their protein sequences were imported from NCBI and used as an additional database («–proteins») (Table S2). All genomes were annotated together using a single command to ensure uniform parameters. The 3LB strain genome was functionally annotated using eggNOG-mapper v2.1.12 [24] and RAST [25], with data from the COG, KEGG, and Gene Ontology databases. [26]. Carbohydrate-active enzymes (CAZymes) were identified with the dbCAN3 metaserver (HMMER tool) [27].

### 4.4 Genomic safety assessment

The antimicrobial activity of strain 3LB was evaluated *in vitro* using the agar well diffusion method (Table S1). The safety assessment of *L. brevis* 3LB strain was performed *in silico* using CARD 4.0.1 (https://card.mcmaster.ca/analyze/rgi, access on 14 September 2025), ResFinder 4.7.2 [28], and NCBI AMRFinderPlus [29], with standard settings used to determine antibiotic resistance. Virulence factors were identified using VirulenceFinder v2.0 (https://cge.food.dtu.dk/services/VirulenceFinder/, available September 14, 2025). PathogenFinder2 v0.5.0 (https://genepi.food.dtu.dk/pathogenfinder, available October 2, 2025) was employed to assess the pathogenic potential of bacterial genomes using genome-based prediction. The PHASTEST software facilitated the detection of prophage regions [30]. PlasmidFinder v2.1 (https://cge.food.dtu.dk/services/PlasmidFinder/, available September 14, 2025) was used to analyze WGS data from our strain to identify and classify plasmid replicons. AntiSMASH v8.0.4 (https://antismash.secondarymetabolites.org/#!/start, available October 13, 2025) was used to detect, annotate, and analyze gene clusters responsible for secondary metabolite biosynthesis in bacterial genomes. CRISPR loci were found using the CRISPRCasFinder database [31].

### 4.5 Data visualization

Data visualization in the form of histograms was accomplished with GraphPad Prism v9.5.1 (GraphPad Software, Boston, MA, USA). Proksee v1.2.0 was employed for the examination of genomic sequences [32].

## Limitations

Not applicable.

## Ethics Statement

The authors have read and complied with the ethical requirements for publication in *Data in Brief*.

## CRediT authorship contribution statement

**Diana Kurmangali:** Writing – original draft, Software, Visualization. **Gulyaim Abitayeva:** Conceptualization, Methodology, Investigation, Writing – review & editing, Funding acquisition. **Zhandarbek Bekshin:** Supervision, Resources.

## Data Availability

NCBIBioSample SAMN52842156 (Original data)
NCBIDDBJ/ENA/GenBank JBSJDQ000000000 (Original data)
NCBIBioProject PRJNA1346845 (Original data)

## Appendix Supplementary materials

Image, application 1
